# Effects of supervised aerobic exercise on cardiorespiratory fitness and patient-reported health outcomes in colorectal cancer patients undergoing adjuvant chemotherapy—a pilot study

**DOI:** 10.1007/s00520-021-06608-9

**Published:** 2021-10-08

**Authors:** Eva M. Zopf, Holger Schulz, Jonas Poeschko, Kerstin Aschenbroich, Thomas Wilhelm, Ernst Eypasch, Elmar Kleimann, Kai Severin, Jutta Benz, Enwu Liu, Wilhelm Bloch, Freerk T. Baumann

**Affiliations:** 1 Department of Molecular and Cellular Sports Medicine, Institute of Cardiology and Sports Medicine, German Sport University Cologne, Cologne, Germany; 2 Mary MacKillop Institute for Health Research, Australian Catholic University, Melbourne, Australia; 3 Praxis Internistischer Onkologie und Hämatologie (Pioh), Frechen, Germany; 4 Augustinian Hospital, Cologne, Germany; 5 Department of Internal Medicine, Center of Integrated Oncology Cologne Bonn, University Hospital of Cologne, Cologne, Germany; 6 St. Vinzenz-Hospital Cologne, Cologne, Germany; 7 Heilig Geist-Hospital Cologne-Longerich, Cologne, Germany; 8 St. Franziskus-Hospital Cologne, Cologne, Germany; 9 MV-Zentrum für Hämatologie und Onkologie, Cologne, Germany; 10 St. Elisabeth-Hospital Cologne-Hohenlind, Cologne, Germany

**Keywords:** Physical activity, Physical fitness, Fatigue, Quality of life, Colorectal neoplasms

## Abstract

**Purpose:**

Colorectal cancer and its treatment are associated with debilitating side effects. Exercise may improve the physical and psychological wellbeing of cancer patients; however, evidence in colorectal cancer patients undergoing adjuvant chemotherapy is limited. This pilot study aimed to explore the effects of supervised aerobic exercise on cardiorespiratory fitness and patient-reported health outcomes in colorectal cancer patients undergoing adjuvant chemotherapy.

**Methods:**

Patients who had undergone curative resection for colorectal cancer (stages II–III) and were scheduled to receive adjuvant chemotherapy were enrolled into this non-randomized controlled trial. Patients in the intervention group (IG) took part in a 6-month supervised aerobic exercise program, while the control group (CG) received usual care. Cardiorespiratory fitness (measured by peak oxygen consumption) was assessed at baseline and 6 months. Fatigue, quality of life, and physical activity levels were additionally assessed at 3 months.

**Results:**

In total, 59 patients (33 in IG vs. 26 in CG) were enrolled into this study. Eighteen patients (9 in IG vs. 9 in CG) dropped out of the study prior to the 6-month follow-up. Significant improvements in cardiorespiratory fitness (*p* = .002) and selected patient-reported health outcomes, such as reduced motivation (*p* = .015) and mental fatigue (*p* = .018), were observed in the IG when compared to the CG.

**Conclusion:**

To our knowledge, this is the first study to investigate the effects of a supervised aerobic exercise program in colorectal cancer patients undergoing adjuvant chemotherapy. The significant and clinically meaningful improvements in CRF warrant further randomized controlled trials to confirm these findings.

**Trials registration:**

German Clinical Trials Register Identifier: DRKS00005793, 11/03/2014, retrospectively registered.

**Supplementary Information:**

The online version contains supplementary material available at 10.1007/s00520-021-06608-9.

## Introduction

Colorectal cancer (CRC) is the third most common cancer worldwide and while survival rates continue to improve, it remains the second most common cause of cancer death [1]. Treatment for CRC is dependent on a variety of factors; however, one of the most common treatment regimens in patients treated with curative intent is surgical resection in combination with adjuvant chemotherapy [2]. Although this treatment approach has proven to extend survival [3], it is often accompanied by debilitating adverse effects including but not limited to pain, fatigue, nausea, diarrhea, mucositis, and peripheral neuropathy [4–6] Furthermore, physical activity levels, physical fitness, and functional capacity have shown to deteriorate during and following CRC treatment, significantly impacting patients’ quality of life (QoL) [7–9]. Poor cardiorespiratory fitness (CRF) has also been associated with increased morbidity [10, 11], and cancer-specific mortality after CRC diagnosis and treatment [12, 13].

The role of physical activity and exercise in the management of cancer and its treatment-related side effects is receiving increasing attention. The recently updated American College of Sports Medicine Exercise Guidelines for Cancer Survivors suggest that exercise has a beneficial effect on a number of cancer-related health outcomes, including fatigue, physical functioning, and health-related QoL [14]. However, the majority of the evidence provided comes from studies involving breast, prostate, or hematological cancer patients. Prospective studies involving CRC patients remain scarce, especially those conducted during CRC treatment [15–17]. In fact, to our knowledge, only five controlled trials have explored the effects of aerobic and resistance exercise specifically in CRC patients undergoing adjuvant chemotherapy. All five studies involved relatively small sample sizes (12–45 patients) and a combined aerobic and resistance exercise program [18–22]. Improvements in fatigue, physical function, role function, pain, nausea, neurotoxicity symptoms, and physical activity levels have been observed [18, 20, 22]. Preliminary evidence also suggests beneficial effects of exercise on chemotherapy completion rates in CRC patients [22].

While aerobic exercise has shown to improve physical fitness, muscle strength, and QoL in breast cancer patients receiving chemotherapy [23, 24], the effects of aerobic exercise in CRC patients undergoing chemotherapy are largely unknown. In a randomized controlled trial involving CRC survivors, some of which were receiving chemotherapy, a home-based aerobic exercise program did not significantly improve health-related QoL, fatigue, or cardiovascular fitness compared to usual care [25]. In a secondary analysis, however, an increase in cardiovascular fitness was associated with improvements in QoL [25]. The effects of a supervised aerobic exercise program in CRC patients undergoing chemotherapy have not yet been explored, although supervised exercise programs are suggested to be more effective than unsupervised/home-based interventions [14, 17].

The CoAktiv study is a non-randomized controlled pilot study, which examined the effects of supervised aerobic exercise training on CRF and patient-reported health outcomes in CRC patients undergoing curative chemotherapy.

## Patients and methods

### Participants

Participants were recruited through three private practices and four hospitals in Cologne, Germany. Eligibility criteria included patients who had undergone curative resection for CRC (stages II–III) and were scheduled to receive adjuvant chemotherapy for at least 6 months. Patients had to be at least 18 years of age and provide written informed consent. Patients were excluded if they presented with severe hypertension or heart disease (NYHA III–IV), ongoing thrombocytopenia (<10000/μ1), congenital or acquired thrombocytopenia or coagulation disorder, respiratory failure, seizure disorder, mental disorder, WHO/ECOG performance status > 2, a physical disability that impeded the use of a cycle ergometer or any other health problem that ruled out regular PA as deemed by their treating physician. Female patients were also excluded if they were pregnant.

### Design

This study was a prospective, 2-armed, non-randomized controlled trial. Potential participants were primarily identified by their treating oncologist and referred to the study coordinator for eligibility screening. Patients that were eligible and willing to participate were invited for baseline testing which included physical fitness testing and the completion of the study questionnaires. To maximize safety, all patients received a medical check-up at baseline, including a resting electrocardiography (ECG), stress ECG, and cardiac ultrasound. Following baseline testing participants were allocated to either the intervention group (IG) or the control group (CG) based on their preference. All patients provided written informed consent prior to participation. Ethical approval was obtained through the ethics committee of the German Sport University Cologne and the study was registered with the German Clinical Trials Registry (DRKS00005793). All procedures conformed to the standards set by the Declaration of Helsinki.

### Exercise intervention

After baseline testing, patients in the IG took part in a 6-month aerobic exercise program while undergoing chemotherapy. Two sessions per week were supervised by an exercise physiologist and included 30 min of cycling on a stationary bicycle. Additionally, patients in the IG were encouraged to complete three 15-min home-based walking sessions per week. The aim was for patients to exercise at a “somewhat hard” to “hard” intensity, measured based on the Borg rating of perceived exertion (RPE) scale [26]. This was expected to correspond to 50–70% of an individual’s peak oxygen uptake (VO2peak), which was derived from the cardiopulmonary exercise test (CPET) at baseline. Hence, the initial training load (watt) for the cycling sessions was set at 50% VO2peak and adjusted following the first training session based on patient’s RPE rating. Taking into account heart rate variability during chemotherapy and given it is common for treatment-related side effects to fluctuate and increase over the course of treatment, the exercise program was progressed and/or modified based on RPE ratings (training rated “light” or “very hard”) and patient wellbeing. Typically, the training load was increased or reduced by 5–10 watts if the training needed to be adjusted. Patients had the option to complete the supervised training sessions at their outpatient clinics (e.g., prior to chemotherapy administration) or at the university gym.

Patients in the CG received usual care, which did not involve any formal exercise advice from the research team; however, patients were not precluded from seeking exercise support independently. Patients in both groups received individual exercise recommendations based on their final CPET after completing the study.

### Assessment of primary and secondary endpoints

The primary endpoint of this study was CRF, measured by VO2peak, which was assessed at baseline (before the exercise intervention/prior to chemotherapy) and at 6 months (after the exercise intervention/post chemotherapy). In order to assess CRF, a CPET on a cycle ergometer was conducted using a modified WHO protocol. The initial work load of 30 watts was increased by 15 watts every 2 min until one of the following conditions occurred: the minute ventilation or ventilation equivalent increased excessively, the ventilation equivalent reached a value between 28 and 30, the respiratory quotient exceeded 1.0, muscular fatigue set in, the patient felt exhausted (RPE ≥ 17), or any complications or symptoms interfered with the testing procedure. During the CPET, relative peak oxygen uptake (VO2peak; in mL/kg/min) as well as the maximal achieved work rate (Wattmax; in watt) and heart rate (HRmax; in bpm) were recorded. As a clear plateau in VO2 may not be achieved before symptom limitation of exercise in clinical populations, VO2peak was defined as the highest oxygen uptake attained during the test. The proportion of patients that achieved a maximal heart rate > 85% of age-predicted maximal heart rate and/or a respiratory exchange ratio (RER) > 1.10 was assessed, to provide an indication of the proportion of patients that gave a maximal effort based on these objective physiological measures [27]. Prior to each CPET, weight and body mass index (BMI) were assessed.

The secondary endpoints included fatigue, QoL, and physical activity levels. These were assessed at baseline, 3 months, and 6 months (after the exercise intervention/post chemotherapy). Fatigue was assessed using the Multidimensional Fatigue Inventory (MFI-20) [28]. QoL was assessed using the European Organization for the Research and Treatment of Cancer Quality of Life Questionnaire (EORTC-QLQ-C30) and its colon cancer–specific module (EORTC-QLQ-CR29) [29, 30]. Physical activity levels (hours per week of leisure time and sport activities) were recorded by means of the Freiburger Questionnaire of Physical Activity [31].

### Statistical analyses

As this study was designed to be a pilot study, a pragmatic approach was taken to determine the sample size. Based on the number of patients treated in the collaborating hospitals and the interest expressed by patients in a prior evaluation, the aim was to recruit 60 patients, 30 per group.

Analyses were performed using IBM SPSS Statistics for Windows, Version 25.0 and included descriptive statistics, independent *t* tests, Fisher’s exact test, and mixed models analysis for repeated measures. Student’s *t* test and Fisher’s exact test were used to compare groups at baseline. Between-group differences in mean changes for individual outcomes were calculated using mixed-model repeated measure analysis. The covariates included in the mixed models approach were group, visit, visit × group and the baseline value for the outcome variables. Participants were treated as random effects (random intercept) and the first-order autoregressive (AR(1)) covariance structure was used. A secondary analysis was conducted, adjusting for age, BMI, and chemotherapy regimen; however, the results were similar and hence the primary analysis is reported in this manuscript. *P* values < 0.05 were considered significant for all analysis. To aid data interpretation, Cohen’s *d* effect sizes were calculated for changes between groups at 3 months (for the questionnaire data) and 6 months/post-intervention (for all data). Based on Cohen’s standard guidelines, effect sizes were defined as follows: *d* = 0 to 0.2 (trivial effect size), *d* = 0.2 to 0.49 (small effect size), *d* = 0.5 to 0.79 (moderate effect size), and *d* = 0.8 and above (strong effect size).

## Results

**Fig. 1:**
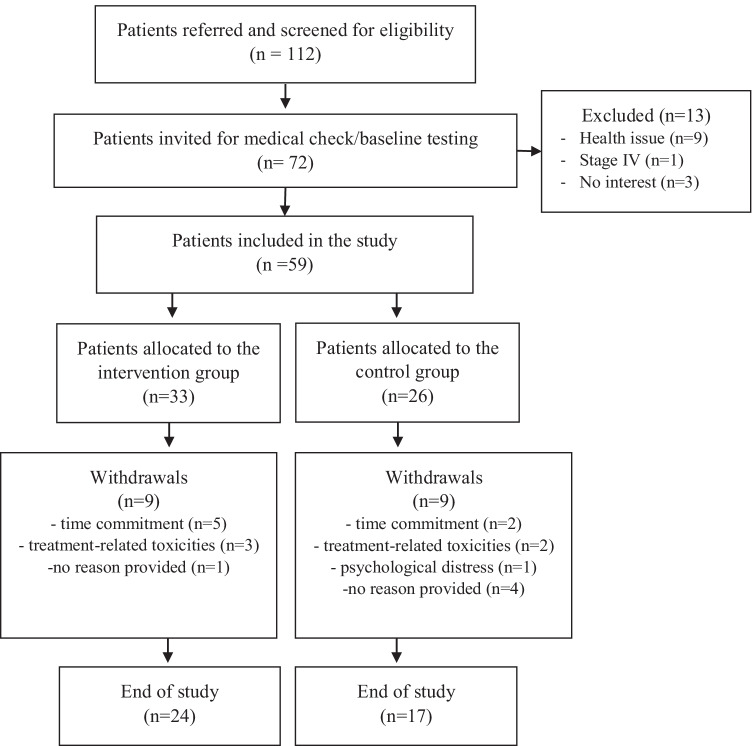
Flow of participants through the study

One-hundred and twelve patients were screened for participation from October 2010 to April 2016. Of the 72 patients (64.3%) that initially expressed interest, 59 patients (52.7%) met all the eligibility criteria and were enrolled into the study. Participants were able to choose between either the exercise intervention or usual care for the duration of their chemotherapy. Eighteen patients (30%) dropped out of the study, nine (27.3%) in the IG and nine (34.6%) in the CG. Reasons for withdrawal included time commitment (*n* = 7), treatment-related toxicities (*n* = 5), and psychological issues (*n* = 1). Six patients did not provide a reason (Fig. 1).

Baseline characteristics of the study participants are presented in Table 1. Thirty-three (55.9%) women and 26 men (44.1%) were included in the study, with the majority of patients being diagnosed with a stage IIIB tumor. There were no statistically significant differences between the two groups at baseline. There were also no statistically significantly differences between participants who withdrew from the study compared to those who completed the study (Online resource 1). No severe exercise-related adverse events were reported in the IG. Available training data (*n* = 23) shows, that on average, patients in the IG that completed the trial attended 67.9% of their exercise sessions (range 39.58–95.83). The attendance rate was 61.9% when including patients that withdrew from the study (range 20.83–95.83). The mean initial training intensity/training load for patients in the IG was set to 63.8% of Vo2peak and 58.6% of Wattmax following patients’ RPE rating after the first training session.

**Table 1:** Baseline characteristics of patients in the intervention and control group

	Intervention group (***n*** = 33)	Control group (***n*** = 26)	***P*** value
Age (years, mean ± SD)	62.1 ± 11.9	57.5 ± 12.1	.146
Body mass index (kg/m^2^, mean ± SD)	24.0 ± 3.8	26.4 ± 5.4	.053
Sex (%)			
-Female	19 (57.6%)	14 (53.8%)	.798
-Male	14 (42.4%)	12 (46.2%)	
Stoma (%)			
-Yes	3 (9.1%)	4 (15.4%)	.688
-No	30 (90.9%)	22 (84.6%)	
Cancer stage (%)			
-II	6 (18.2%)	3 (11.5%)	.858
-III	26 (78.8%)	22 (84.6%)	
-n.a	1 (3.0%)	1 (3.8%)	
Chemotherapy regime (%)			
-FOLFOX	16 (48.5%)	17 (65.4%)	.645
-XELODA	8 (24.2%)	4 (15.4%)	
-XELOX	8 (24.2%)	4 (15.4%)	
-n.a	1 (3.0%)	1 (3.8%)	

*FOLFOX*, oxaliplatin, fluorouracil, folinic acid; *XELODA*, capecitabine; *XELOX*, oxaliplatin and capecitabine

### Primary outcome

The effect of the exercise intervention on CRF is summarized in Table 2 and Online resource 3. Relative VO2peak and Wattmax improved in the IG compared to the CG (mean difference 4.11 ml/kg/min; 95% CI, 1.52–6.71; *p* = 0.002 and mean difference 16.14 W; 95% CI, 5.71–26.57; *p* = 0.003, respectively). Relative VO2peak and Wattmax improved by 3.57 ml/kg/min (95% CI, 1.90–5.25; *p* < 0.001) and 22.20 W (95% CI, 15.55–28.85; *p* < 0.001), respectively, from baseline to post-intervention within the IG. Cohen’s *d* effect sizes for VO2peak and Wattmax were moderate (0.53 and 0.51, respectively) at post-intervention. Based on achieving a maximal heart rate > 85% of age-predicted maximal heart rate and/or a RER > 1.10, 86% and 95% of patients gave a maximal effort in their CPET at baseline and post-intervention, respectively. Weight and BMI increased significantly within both groups from baseline to post-intervention, with no significant difference between groups.

**Table 2:** Effects of a 6-month aerobic exercise intervention on cardiorespiratory fitness, maximal work rate, maximal heart rate, body weight, and body mass index in colorectal cancer patients undergoing adjuvant chemotherapy

		Baseline	Post-intervention		Between-group difference from baseline to post-intervention	
		Mean^a^ (*SE*)	Mean^a^ (*SE*)	*P* value^b^	Mean difference (95% CI)	*P* value
VO2peak relative (ml/kg/min)	IG	21.67 (0.55)	25.24 (0.63)	** < .001**	4.11 (1.52; 6.71)	**.002**
	CG	21.71 (0.61)	21.17 (0.79)	.587		
Wattmax (watt)	IG	113.22 (2.17)	135.42 (2.50)	** < .001**	16.14 (5.71; 26.57)	**.003**
	CG	113.45 (2.45)	119.51 (3.17)	.136		
HRmax (bpm)	IG	151.57 (1.67)	151.76 (1.92)	.945	4.20 (− 4.23; 12.63)	.323
	CG	151.65 (1.89)	147.64 (2.43)	.220		
Weight (kg)	IG	73.95 (0.43)	75.62 (0.50)	**.015**	− 0.70 (− 2.77; 1.37)	.503
	CG	73.77 (0.49)	76.13 (0.62)	**.004**		
BMI (kg/m^2^)	IG	25.24 (0.14)	25.81 (0.16)	**.010**	− 0.18 (− 0.86; 0.49)	.588
	CG	25.17 (0.16)	25.94 (0.21)	**.005**		

*BMI*, body mass index; *CG*, control group; *CI*, confidence interval; *HRmax*, maximal heart rate; *IG*, intervention group; *SE*, standard error; *VO2peak*, peak oxygen uptake; *Wattmax*, maximal work rate

^a^Least square mean and standard error following mixed-model repeated measure analysis

^b^*P* value for changes within groups from baseline to post-intervention

### Secondary outcomes

The effects of the exercise intervention on patient-reported health outcomes and physical activity levels are summarized in Table 3, Online resource 2, and Online resource 3. With regard to the MFI, differences between groups in favor of the IG were observed for reduced motivation at 3 months and 6 months (mean difference − 2.07; 95% CI, − 3.73–-0.41; *p* = 0.015, Cohen’s *d* effect size 0.29 and mean difference − 1.94; 95% CI, − 3.79–-0.09; *p* = 0.040, Cohen’s *d* effect size 0.36, respectively) and mental fatigue at 3-months (mean difference − 2.23; 95% CI, − 4.06–-0.40, *p* = 0.018, Cohen’s *d* effect size 0.36). Group differences in fatigue were primarily apparent at 3 months, with general fatigue, reduced motivation, and mental fatigue increasing by 2.34 (95% CI, 0.68–4.01; *p* = 0.003), 1.80 (95% CI, 0.25–3.35; *p* = 0.017), 1.99 (95% CI, 0.28–3.70; *p* = 0.017), respectively, in the CG from baseline to 3 months.

**Table 3:** Effects of a 6-month aerobic exercise intervention on fatigue in colorectal cancer patients undergoing adjuvant chemotherapy

		Baseline	3 months		Post-intervention		Between-group difference from baseline to 3-months		Between-group difference from baseline to post-intervention	
		Mean^a^ (*SE*)	Mean^a^ (*SE*)	*P* value^b^	Mean^a^ (*SE*)	*P* value^c^	Mean difference (95% CI)	*P* value	Mean difference (95% CI)	*P* value
MFI										
General fatigue	IG	9.44 (.45)	10.63 (.51)	.121	10.22 (.54)	.751	− 1.15 (− 2.92; 0.63)	.203	1.32 (− 0.72; 3.36)	.203
	CG	9.66 (.51)	12.00 (.60)	**.003**	9.11 (.63)	1.000				
Physical fatigue	IG	10.67 (.49)	9.74 (.55)	.570	9.71 (.58)	.624	− 1.83 (− 3.99; 3.33)	.097	− .75 (− 3.03; 1.53)	.515
	CG	10.65 (.55)	11.55 (.66)	.847	10.45 (.68)	1.000				
Reduced activity	IG	10.11 (.51)	10.37 (.57)	1.000	9.64 (.60)	1.000	− .08 (-2.14; 1.98)	.940	.71 (− 1.61; 3.02)	.546
	CG	9.92 (.57)	10.25 (.68)	1.000	8.74 (.70)	.553				
Reduced motivation	IG	8.14 (.41)	7.87 (.45)	1.000	6.83 (.49)	.103	− 2.07 (− 3.73; − 0.41)	**.015**	− 1.94 (− 3.79; − 0.09)	**.040**
	CG	7.67 (.46)	9.47 (.55)	**.017**	8.30 (.57)	1.000				
Mental fatigue	IG	7.46 (.39)	7.22 (.44)	1.000	7.21 (.47)	1.000	− 2.23 (-4.06; -0.40)	**.018**	− 0.06 (− 1.89; 1.77)	.950
	CG	7.15 (.44)	9.14 (.53)	**.017**	6.96 (.54)	1.000				

*CG*, control group; *CI*, confidence interval; *IG*, intervention group; *MFI*, Multidimensional Fatigue Inventory; *SE*, standard error

^a^Least square mean and standard error following mixed-model repeated measure analysis

^b^*P* value for changes within groups from baseline to 3 months

^c^*P* value for changes within groups from baseline to post-intervention

With regard to the EORTC-QLQ C30, a between-group difference was observed in role function in favor of the IG at 3 months (mean difference 19.64; 95% CI, 3.47–35.81; *p* = 0.018; Cohen’s *d* effect size 0.36). Nausea/vomiting increased within both groups from baseline to 3 months (IG: mean difference − 10.01; 95% CI, − 16.43–-3.60; *p* = 0.001; CG: mean difference − 10.33; 95% CI, − 17.87–-2.80; *p* = 0.004). In relation to the EORTC-QLQ CR29, an improvement in favor of the IG was observed for urinary frequency at 3 months (mean difference − 13.21; 95%, CI − 24.48–-1.93; *p* = 0.022, Cohen’s *d* effect size 0.37) as well as blood and mucus in the stool at 3 months and 6 months (mean difference − 12.24; 95%, CI, − 17.87–-6.61, *p* < 0.001, Cohen’s *d* effect size 0.52 and mean difference − 10.63; 95% CI, − 17.23–-4.03; *p* = 0.002, Cohen’s *d* effect size 0.42, respectively). Significant time effects were observed for dysuria, abdominal pain, buttock pain, dry mouth, hair loss, trouble with taste, anxiety, and sore skin around anus/stoma.

No significant differences within or between groups were observed for self-reported leisure time and sport activity levels at 3 months or 6 months.

## Discussion

A 6-month supervised aerobic exercise program delivered to CRC patients undergoing adjuvant chemotherapy led to significant improvements in CRF and selected patient-reported health outcomes, such as reduced motivation and mental fatigue, compared to usual care. To our knowledge, this is the first study to demonstrate significant improvements in CRF following a supervised aerobic exercise intervention during adjuvant chemotherapy in CRC patients. These results are clinically meaningful given poor CRF has been associated with higher symptom burden and morbidity [10, 11, 25, 32, 33] as well as an increased risk of overall and cancer-specific mortality [12, 13].

Determining the effects of exercise on CRF in CRC patient undergoing adjuvant chemotherapy has been limited by a small number of studies and inconclusive results [18–21]. Our study, however, supports the findings of a recent meta-analysis that included five studies involving CRC patients undergoing chemotherapy in a subgroup analysis and revealed significant effects of exercise on aerobic fitness compared with usual care [17]. Notably, however, none of the studies included in the subgroup analysis assessed a supervised aerobic exercise program, not all patients in the included studies received adjuvant chemotherapy, and only one study assessed VO2peak, the gold standard for assessing CRF. Furthermore, the meta-analytic findings suggest a larger benefit for aerobic fitness for unsupervised interventions in CRC survivors, while we observed a significant effect of supervised exercise [17]. Hence, further randomized controlled trials are warranted to confirm the effects of supervised aerobic exercise on CRF in CRC patients receiving adjuvant chemotherapy.

In our study, CRF improved by 3.57 mL·kg^−1^·min^−1^ (16.5%) in the IG, compared to a decrease of 0.54 mL·kg^−1^·min^−1^ (− 2.6%) in the CG. This is an important clinical outcome, given each 1 mL·kg^−1^·min^−1^ increase in CRF has been associated with a ~ 11, 15, and 16% reduction in all-cause, cardiovascular disease, and cancer mortality, respectively, in healthy men and women [34]. While further investigations in CRC patients are required, an increase in physical activity by at least 10 metabolic equivalent task-hour per week (MET-h/week), which corresponds to the exercise prescribed in this study, has been associated with a 28% lower risk of mortality after CRC diagnosis [35]. Our findings and these observations further support the call for prospective studies exploring the effects of exercise and improvements in CRF on key clinical outcomes, such as cancer recurrence and mortality.

The effect of exercise on QoL and fatigue in CRC patients undergoing adjuvant chemotherapy has been inconclusive. While a recent meta-analysis of studies involving CRC patients pre-treatment, during treatment and post-treatment found significant effects of exercise compared to usual care on QoL and fatigue, a subgroup analysis suggested larger effects on QoL post-treatment [17]. In line with the meta-analytic findings and previous studies in CRC patients undergoing chemotherapy, we only observed improvements in selected QoL domains during adjuvant chemotherapy and the effect sizes were mostly small. Yet, the significant improvement in role function that we observed in the IG has also been described by Lin et al., who prescribed a supervised aerobic and resistance exercise program to CRC patients undergoing chemotherapy [18]. While studies in CRC survivors suggest mixed mode exercise interventions or higher doses of aerobic exercise (up to 300 min·wk^−1^) may improve health-related QoL more effectively [17, 36], this requires further investigation in CRC patients undergoing chemotherapy. In this study, we focused on aerobic exercise rather than a multimodal intervention, as CRF was our primary outcome and when the study was designed, aerobic exercise showed more beneficial effects on fatigue than other exercise modalities.

Irrespective of the exercise mode or intensity, meta-analyses in cancer patients, including those with CRC and those undergoing chemotherapy, suggest supervised exercise interventions are more effective at improving QoL and fatigue [17, 37, 38]. The significant improvements we observed in reduced motivation and mental fatigue are in line with these findings. Similarly, Van Vulpen et al. found that CRC patients undergoing chemotherapy experience less fatigue when participating in a supervised exercise program involving two supervised sessions per week [20]. Notably, however, they observed improvement in physical and general fatigue, while we observed improvements in reduced motivation and mental fatigue. While meta-analytic data in breast cancer patients undergoing adjuvant treatment suggests that physical fatigue is most sensitive to physical exercise [39], further randomized controlled studies in CRC patients undergoing adjuvant chemotherapy are required to explore what exercise settings, modes and intensities are most effective at improving the different dimensions of fatigue. Importantly, while effect sizes were small in our study, they are comparable to previous exercise intervention studies during chemotherapy [39] and may still be perceived as clinically relevant. The minimal clinically important difference for changes in fatigue as measured by the MFI has been identified as two points in cancer patients undergoing treatment [40], which corresponds to the differences we observed in general fatigue (within CG), reduced motivation, and mental fatigue.

Apart from fatigue, surgical resection in combination with adjuvant chemotherapy is often associated with other debilitating adverse effects that negatively affect QoL of CRC patients, such as pain, nausea, diarrhea, and mucositis [4–6]. In our study, we observed a significant group effect for urinary frequency as well as blood and mucus in the stool following the supervised aerobic exercise program. Whether this is related to the exercise intervention requires further investigations. Notably, the blood and mucus in the stool scale of the EORTC-QLQ-CR29 has been shown to be only moderately reliable [30, 41], and our results were driven by three patients who reported high scores (> 50) at baseline, two of which were in the CG and withdrew from the study for unrelated issues. Although we observed favorable within-group changes in abdominal pain and dysuria in the IG, while flatulence increased in the CG, preliminary data suggests that more specific exercise modalities, such as pelvic floor muscle training, may be more effective at improving some of the treatment-related side effects of CRC surgery, such as bowel function [42]. Yet, combined aerobic and resistance programs have shown some promise in decreasing pain, nausea, and neurotoxicity symptoms in CRC patients undergoing adjuvant treatment [18, 22]. Given disease- and treatment-related side effects have shown to significantly impact physical activity levels and QoL in CRC patients undergoing adjuvant treatment [8], further investigations focusing on these outcomes would be worthwhile.

While this study underpins that exercise training is feasible and effective in CRC patients undergoing treatment, there are some limitations. The main limitation of the current study is the study design. We chose a non-randomized controlled study design because we anticipated recruitment and compliance challenges based on previous studies conducted with CRC patients. And in fact, compared to the average recruitment rate of 38% (range: 4–91%) in randomized controlled trials involving CRC patients [17], we enrolled 53% of screened patients. While recruitment was slow, the strategy of having clinicians talk to patients about the study and refer them directly may have contributed to this participation rate. The impact of clinician referral on exercise participation has been described previously [43]. The number of patients that did not complete the study (30%) was similar to other studies [20, 25]. Despite the lack of randomization, the two groups were comparable at baseline with regard to patient characteristics and all study outcomes. Nevertheless, given a pragmatic approach was taken to determine the sample size and group allocation was based on patient preference, the findings of this pilot study need to be interpreted with caution and further RCTs are required to confirm the results. This may also be warranted given multiple comparison adjustment was not applied in the present analysis. Furthermore, we did not have access to all medical records to accurately report the actual chemotherapy plan that patients completed (incl. actual duration and dose). A strength of this study is its relatively large sample size and long (supervised) intervention, compared to other studies involving CRC patients undergoing adjuvant chemotherapy. While challenges in engaging CRC patients in exercise trials are commonly reported, we almost managed to reach our target sample size of 60 patients and enroll and assess all patients prior to initiating chemotherapy. Further strengths of our trial that likely contributed to its feasibility were that patients had the option of exercising at their outpatient clinic (e.g., prior to chemotherapy administration) and all training locations were easily accessible (free parking and/or access via public transport). Together with providing supervision, a convenient exercise location has been identified as an important facilitator when it comes to exercise participation and intervention success [14, 43].

## Conclusion

Although exercise guidelines recommend CRC patients return to daily activities and physical exercise shortly after surgery and continue to be physical active during nonsurgical treatment [44], studies conducted during adjuvant chemotherapy for CRC remain scarce. To our knowledge, this is the first study to investigate the effects of a supervised aerobic exercise program in CRC patients undergoing adjuvant chemotherapy. The significant improvements in CRF compared to usual care are promising and clinically meaningful, and hence warrant further randomized controlled trials to confirm these findings and further explore the optimal exercise modes and intensities to improve the health and wellbeing of CRC patients undergoing adjuvant chemotherapy.

## Supplementary Information

Below is the link to the electronic supplementary material.

Supplementary file1 (DOCX 17 KB)

Supplementary file2 (DOCX 39 KB)

Supplementary file3 (DOCX 34.0 KB)
