# Supplementary material for: Effects of supervised aerobic exercise on cardiorespiratory fitness and patient-reported health outcomes in colorectal cancer patients undergoing adjuvant chemotherapy—a pilot study
Source: Support Care Cancer. 2021 Oct 8;30(3):1945–55. doi: 10.1007/s00520-021-06608-9 (PMC8795052; doi:10.1007/s00520-021-06608-9)
Supplement: Supplementary file 1 — Supplementary file1 (DOCX 17 KB) [file 520_2021_6608_MOESM1_ESM.docx]

**Effects of supervised aerobic exercise on cardiorespiratory fitness and patient-reported health outcomes in colorectal cancer patients undergoing adjuvant chemotherapy – a pilot study**

Supportive Care in Cancer

Eva M Zopf, Holger Schulz, Jonas Poeschko, Kerstin Aschenbroich, Thomas Wilhelm, Ernst Eypasch, Elmar Kleimann, Kai Severin, Jutta Benz, Enwu Liu, Wilhelm Bloch, Freerk T Baumann

Corresponding author: PD Dr Freerk T Baumann, Department of Internal Medicine, Center of Integrated Oncology Cologne Bonn, University Hospital of Cologne, Cologne, Germany, [freerk.baumann@uk-koeln.de](mailto:freerk.baumann@uk-koeln.de)

**Online resource 1** Baseline characteristics of patients who completed the study and those who dropped out of the study

|  | **Non-Dropouts**  **(n=41)** | **Dropouts**  **(n=18)** | | **p-value** |
| --- | --- | --- | --- | --- |
| **Age** (years, mean ± SD) | 60.9 ± 11.7 | | 58.1 ± 13.2 | .452 |
| **Body Mass Index** (kg/m^2^, mean ± SD) | 25.5 ± 4.3 | | 24.1 ± 5.5 | .341 |
| **Sex** (%)   - Female   Male | 24 (58.5%)  17 (41.5%) | | 9 (50%)  9 (50%) | .580 |
| **Stoma** (%)   - Yes - No | 4 (9.8%)  37 (90.2%) | | 3 (16.7%)  15 (83.3%) | .664 |
| **Cancer stage** (%)   - II - III - n.a | 6 (14.6%)  33 (80.5%)  2 (4.9%) | | 3 (16.7%)  15 (83.3%)  0 (0%) | 1.000 |
| **Chemotherapy Regime** (%)   - FOLFOX - XELODA - XELOX - n.a. | 22 (53.6%)  10 (24.4%)  7 (17.1%)  2 (4.9%) | | 11 (61.1%)  2 (11.1%)  5 (27.8%)  0 (0%) | .475 |

Abbreviations: FOLFOX, Oxaliplatin, fluorouracil, folinic acid; XELODA, Capecitabine; XELOX, Oxaliplatin & Capecitabine
