# Supplementary material for: Effects of supervised aerobic exercise on cardiorespiratory fitness and patient-reported health outcomes in colorectal cancer patients undergoing adjuvant chemotherapy—a pilot study
Source: Support Care Cancer. 2021 Oct 8;30(3):1945–55. doi: 10.1007/s00520-021-06608-9 (PMC8795052; doi:10.1007/s00520-021-06608-9)
Supplement: Supplementary file 2 — Supplementary file2 (DOCX 39 KB) [file 520_2021_6608_MOESM2_ESM.docx]

**Effects of supervised aerobic exercise on cardiorespiratory fitness and patient-reported health outcomes in colorectal cancer patients undergoing adjuvant chemotherapy – a pilot study**

Supportive Care in Cancer

Eva M Zopf, Holger Schulz, Jonas Poeschko, Kerstin Aschenbroich, Thomas Wilhelm, Ernst Eypasch, Elmar Kleimann, Kai Severin, Jutta Benz, Enwu Liu, Wilhelm Bloch, Freerk T Baumann

Corresponding author: PD Dr Freerk T Baumann, Department of Internal Medicine, Center of Integrated Oncology Cologne Bonn, University Hospital of Cologne, Cologne, Germany, [freerk.baumann@uk-koeln.de](mailto:freerk.baumann@uk-koeln.de)

**Online resource 2** Effects of a 6-month aerobic exercise intervention on quality of life and physical activity levels in colorectal cancer patients undergoing adjuvant chemotherapy

|  | | Baseline | 3-months | | Post-intervention | | Between-group difference from baseline to 3-months | | Between-group difference from baseline to post-intervention | |
| --- | --- | --- | --- | --- | --- | --- | --- | --- | --- | --- |
|  |  | Mean^a^ (SE) | Mean^a^ (SE) | P value^b^ | Mean (SE) | P value^c^ | Mean difference (95% CI) | P value | Mean difference (95% CI) | P value |
| EORTC-QLQ C30 | | | | | | | | | | |
| Global health status/QoL | IG | 61.26 (2.64) | 61.69 (2.96) | 1.000 | 67.68 (3.09) | .339 | 1.31  (-9.96; 12.57) | .819 | -2.76  (-14.94; 9.43) | .655 |
|  | CG | 61.66 (2.97) | 60.77 (3.56) | 1.000 | 70.83 (3.67) | .155 |  |  |  |  |
| Physical function | IG | 82.54 (2.41) | 80.81 (2.70) | 1.000 | 82.22 (2.82) | 1.000 | 6.16  (-3.70; 16.01) | .218 | 1.95  (-9.04; 12.93) | .727 |
|  | CG | 82.47 (2.72) | 74.58 (3.24) | .120 | 80.21 (3.36) | 1.000 |  |  |  |  |
| Role function | IG | 55.77 (3.76) | 70.64 (4.23) | **.018** | 75.36 (4.42) | **.003** | 19.64  (3.47; 35.81) | **.018** | 11.84  (-5.59; 29.27) | .181 |
|  | CG | 57.31 (4.24) | 52.53 (5.09) | 1.000 | 65.06 (5.25) | .743 |  |  |  |  |
| Emotional function | IG | 70.24 (2.79) | 74.43 (3.12) | .751 | 80.06 (3.26) | .058 | 6.16  (-4.95; 17.27) | .274 | -0.82  (-13.42; 11.78) | .898 |
|  | CG | 68.21 (3.17) | 66.24 (3.73) | 1.000 | 78.84 (3.88) | .090 |  |  |  |  |
| Cognitive function | IG | 81.78 (3.04) | 85.93 (3.42) | 1.000 | 89.31 (3.57) | .324 | 2.65  (-10.62; 15.91) | .693 | 0.34  (-13.78; 14.45) | .962 |
|  | CG | 84.90 (3.42) | 86.40 (4.11) | 1.000 | 92.09 (4.24) | .558 |  |  |  |  |
| Social function | IG | 71.50 (2.86) | 75.86 (3.20) | .786 | 79.36 (3.35) | .211 | 10.58  (-1.25; 22.41) | .079 | 4.47  (-8.62; 17.56) | .500 |
|  | CG | 71.33 (3.22) | 65.10 (3.84) | .520 | 74.72 (3.98) | 1.000 |  |  |  |  |
| Fatigue | IG | 36.25 (3.29) | 37.49 (3.68) | 1.000 | 30.25 (3.85) | .679 | -7.21  (-20.69; 6.27) | .291 | 1.26  (-13.80; 16.33) | .868 |
|  | CG | 37.91 (3.71) | 46.36 (4.41) | .317 | 30.64 (4.58) | .631 |  |  |  |  |
| Nausea/vomiting | IG | 2.30 (1.92) | 12.31 (2.15) | **.001** | 7.39 (2.24) | .246 | -0.32  (-8.38; 7.74) | .937 | 4.37  (-4.45; 13.20) | .329 |
|  | CG | 2.85 (2.16) | 13.19 (2.59) | **.004** | 3.57 (2.66) | 1.000 |  |  |  |  |
| Pain | IG | 25.67 (4.04) | 23.47 (4.55) | 1.000 | 19.25 (4.74) | .912 | 1.95  (-16.20; 20.09) | .832 | .84  (-18.04; 19.71) | .930 |
|  | CG | 27.17 (4.55) | 23.03 (5.47) | 1.000 | 19.92 (5.63) | .952 |  |  |  |  |
| Dyspnea | IG | 19.13 (3.26) | 18.95 (3.64) | 1.000 | 19.87 (3.80) | 1.000 | -6.37  (-19.59; 6.85) | .341 | 2.96  (-11.82; 17.73) | .693 |
|  | CG | 23.06 (3.71) | 29.26 (4.36) | .677 | 20.85 (4.51) | 1.000 |  |  |  |  |
| Insomnia | IG | 26.79 (3.93) | 22.23 (4.41) | 1.000 | 28.15 (4.60) | 1.000 | -3.13  (-19.77; 13.50) | .709 | 14.22  (-3.90; 32.35) | .123 |
|  | CG | 28.40 (4.43) | 26.97 (5.29) | 1.000 | 15.53 (5.47) | .200 |  |  |  |  |
| Appetite loss | IG | 11.80 (3.07) | 11.34 (3.44) | 1.000 | 12.63 (3.59) | 1.000 | -9.04  (-22.60; 4.52) | .189 | 1.54  (-12.70; 15.78) | .831 |
|  | CG | 10.47 (3.45) | 19.05 (4.15) | .307 | 9.76 (4.27) | 1.000 |  |  |  |  |
| Constipation | IG | 8.01 (2.52) | 6.71 (2.79) | 1.000 | 7.67 (2.93) | 1.000 | -1.17  (-10.54; 8.21) | .805 | -2.29  (-13.37; 8.78) | .683 |
|  | CG | 7.30 (2.90) | 7.17 (3.42) | 1.000 | 9.26 (3.59) | 1.000 |  |  |  |  |
| Diarrhea | IG | 23.04 (3.43) | 25.72 (3.83) | 1.000 | 19.20 (4.00) | 1.000 | -1.79  (-15.45; 11.87) | .795 | -2.78  (-18.26; 12.69) | .722 |
|  | CG | 23.13 (3.87) | 27.60 (4.58) | 1.000 | 22.06 (4.77) | 1.000 |  |  |  |  |
| Financial difficulties | IG | 17.10 (3.15) | 23.54 (3.49) | .282 | 17.63 (3.66) | 1.000 | 11.30  (-0.41; 23.01) | .058 | 3.20  (-10.54; 16.94) | .646 |
|  | CG | 17.40 (3.54) | 12.54 (4.16) | .851 | 14.73 (4.36) | 1.000 |  |  |  |  |
| EORTC-QLQ CR29 | | | | | | | | | | |
| Urinary frequency | IG | 42.71 (3.05) | 28.49 (3.42) | **.001** | 35.43 (3.54) | .289 | -13.21  (-24.48; -1.93) | **.022** | -2.85  (-16.06; 10.36) | .670 |
|  | CG | 44.45 (3.44) | 43.44 (4.02) | 1.000 | 40.03 (4.21) | 1.000 |  |  |  |  |
| Blood and mucus in stool | IG | 7.46 (1.54) | .47 (1.72) | **.001** | .218 (1.77) | .**003** | -12.24  (-17.87; -6.61) | **<.001** | -10.63  (-17.23; -4.03) | **.002** |
|  | CG | 5.77 (1.72) | 11.02 (2.02) | **.050** | 9.15 (2.11) | .552 |  |  |  |  |
| Body image | IG | 83.42 (2.85) | 77.72 (3.25) | .410 | 75.76 (3.33) | .219 | -5.11  (-16.70; 6.47) | .383 | -10.35  (-23.24; 2.55) | .115 |
|  | CG | 80.71 (3.20) | 80.12 (3.84) | 1.000 | 83.40 (3.40) | 1.000 |  |  |  |  |
| Urinary incontinence | IG | 3.29 (2.40) | 1.78 (2.72) | 1.000 | 7.56 (2.86) | .678 | -0.05  (-9.16; 9.05) | .991 | 1.66  (-8.96; 12.27) | .758 |
|  | CG | 4.13 (2.70) | 2.67 (3.19) | 1.000 | 6.74 (3.34) | 1.000 |  |  |  |  |
| Dysuria | IG | 5.71 (1.50) | 4.03 (1.72) | 1.000 | -.21 (1.80) | **.038** | -0.03  (-7.01; 6.94) | .993 | -1.44  (-8.49; 5.60) | .686 |
|  | CG | 5.27 (1.69) | 3.62 (2.03) | 1.000 | .80 (2.09) | .294 |  |  |  |  |
| Abdominal pain | IG | 22.00 (3.00) | 7.62 (3.46) | **.004** | 9.88 (3.54) | **.029** | -7.72  (-21.04; 5.61) | .253 | -3.11  (-17.14; 10.92) | .662 |
|  | CG | 19.94 (3.38) | 13.28 (4.07) | .577 | 10.92 (4.23) | .287 |  |  |  |  |
| Buttock pain | IG | 14.53 (2.72) | 12.20 (3.09) | 1.000 | 11.35 (3.18) | 1.000 | 2.81  (-8.12; 13.75) | .611 | 10.66  (-1.64; 22.95) | .089 |
|  | CG | 15.62 (3.07) | 10.48 (3.64) | .665 | 1.78 (3.78) | .**012** |  |  |  |  |
| Bloated feeling | IG | 18.02 (3.58) | 19.21 (4.11) | 1.000 | 13.30 (4.19) | 1.000 | 2.13  (-13.66; 17.91) | .790 | -5.19  (-21.84; 11.45) | .538 |
|  | CG | 16.00 (4.04) | 15.06 (4.84) | 1.000 | 16.46 (4.98) | 1.000 |  |  |  |  |
| Dry mouth | IG | 22.04 (3.92) | 32.52 (4.46) | .153 | 22.35 (4.56) | 1.000 | -6.61  (-22.78; 9.55) | .419 | -2.63  (-20.43; 15.16) | .770 |
|  | CG | 19.83 (4.38) | 36.93 (5.26) | **.020** | 22.78 (5.45) | 1.000 |  |  |  |  |
| Hair loss | IG | 2.10 (3.48) | 20.31 (3.96) | **<.001** | 21.90 (4.07) | **.001** | -3.42  (-17.49; 10.64) | .630 | 4.58  (-11.12; 20.27) | .565 |
|  | CG | 2.18 (3.92) | 23.82 (4.78) | **<.001** | 17.41 (4.83) | **.038** |  |  |  |  |
| Trouble with taste | IG | 5.90 (4.53) | 39.74 (5.18) | **<.001** | 33.49 (5.32) | **<.001** | 4.75  (-14.59; 24.08) | .627 | 16.17  (-4.58; 36.93) | .126 |
|  | CG | 4.61 (5.11) | 33.71 (6.45) | **.001** | 16.03 (6.31) | .460 |  |  |  |  |
| Anxiety | IG | 48.75 (3.89) | 63.37 (4.44) | **.020** | 62.83 (4.55) | .052 | 6.06  (-9.97; 22.09) | .455 | -5.11  (-22.87; 12.65) | .570 |
|  | CG | 46.63 (4.39) | 55.19 (5.22) | .496 | 65.82 (5.41) | **.017** |  |  |  |  |
| Weight | IG | 75.24 (4.24) | 80.04 (4.81) | 1.000 | 74.23 (4.93) | 1.000 | 7.92  (-9.63; 25.47) | .372 | 2.03  (-17.25; 21.31) | .835 |
|  | CG | 76.82 (4.74) | 73.70 (5.67) | 1.000 | 73.78 (5.89) | 1.000 |  |  |  |  |
| Flatulence | IG | 11.21 (3.66) | 15.58 (4.21) | 1.000 | 12.94 (4.25) | 1.000 | -3.89  (-18.64; 11.26) | .625 | -15.90  (-32.88; 1.08) | .066 |
|  | CG | 10.03 (4.27) | 18.09 (5.01) | .493 | 27.65 (5.36) | **.027** |  |  |  |  |
| Faecal incontinence | IG | 6.20 (2.65) | 4.263 (3.06) | 1.000 | 2.87 (3.06) | 1.000 | -2.28  (-14.60; 10.02) | .713 | 1.10  (-11.50; 13.71) | .863 |
|  | CG | 9.37 (3.06) | 9.71 (3.64) | 1.000 | 4.93 (3.87) | 1.000 |  |  |  |  |
| Sore skin around anus or stoma | IG | 8.30 (3.22) | 19.52 (3.74) | **.040** | 10.00 (3.76) | 1.000 | -2.02  (-15.70; 11.66) | .770 | 4.76  (-10.42; 19.95) | .536 |
|  | CG | 8.11 (3.77) | 21.34 (4.45) | **.041** | 5.04 (4.75) | 1.000 |  |  |  |  |
| Stool frequency | IG | 22.06 (2.61) | 21.98 (3.04) | 1.000 | 19.20 (3.06) | 1.000 | -3.19  (-14.13; 7.74) | .563 | -4.04  (-16.31; 8.22) | .515 |
|  | CG | 22.56 (3.07) | 25.68 (3.61) | 1.000 | 23.75 (3.86) | 1.000 |  |  |  |  |
| Embarrassment by defaecation pattern or stoma | IG | 6.08 (2.45) | 9.88 (2.82) | .686 | 10.25 (2.86) | .735 | 2.01  (-7.63; 11.66) | .680 | 6.62  (-4.56; 17.80) | .244 |
|  | CG | 6.60 (2.88) | 8.38 (3.35) | 1.000 | 4.15 (3.60) | 1.000 |  |  |  |  |
| Sexual interest (male) | IG | 55.78 (5.21) | 40.73 (5.83) | .100 | 42.86 (6.82) | .382 | -10.32  (-30.81; 10.17) | .315 | -23.42  (-47.97; 1.13) | .061 |
|  | CG | 54.45 (5.62) | 49.72 (6.45) | 1.000 | 64.96 (7.30) | .739 |  |  |  |  |
| Impotence (male) | IG | 23.57 (6.93) | 35.41 (7.81) | .747 | 42.86 (8.74) | .257 | 14.35  (-16.00; 44.69) | .345 | 16.50  (-15.91; 48.92) | .312 |
|  | CG | 28.46 (7.48) | 25.95 (8.67) | 1.000 | 31.25 (9.27) | 1.000 |  |  |  |  |
| Sexual interest (female) | IG | 21.51 (5.20) | 24.93 (5.88) | 1.000 | 27.66 (5.69) | 1.000 | 3.27  (-21.16; 27.71) | .789 | -12.53  (-36.97; 11.91) | .310 |
|  | CG | 19.79 (6.11) | 19.95 (7.34) | 1.000 | 38.47 (7.34) | .163 |  |  |  |  |
| Dyspareunia (female) | IG | 3.44 (4.78) | 11.85 (5.39) | .534 | 7.53 (5.26) | 1.000 | 2.76  (-19.67; 25.19) | .805 | -6.19  (-28.98; 16.61) | .589 |
|  | CG | 3.01 (5.94) | 8.66 (8.47) | 1.000 | 13.28 (7.44) | .795 |  |  |  |  |
| Freiburger Questionnaire of Physical Activity | | | | | | | | | | |
| Leisure time & sport activity (h/week) | IG | 3.48 (1.12) | 7.41 (1.26) | .069 | 3.90 (1.31) | 1.000 | 2.87  (-2.34; 8.07) | .277 | -2.53  (-7.77; 2.71) | .341 |
|  | CG | 3.32 (1.26) | 4.38 (1.52) | 1.000 | 6.27 (1.57) | .432 |  |  |  |  |

Abbreviations: CG control group, CI confidence interval, EORTC-QLQ C30 European Organization for the Research and Treatment of Cancer Quality of Life Questionnaire, EORTC-QLQ CR29 colon cancer specific module, IG intervention group, QoL Quality of life, SE standard error

^a^ least square mean and standard error following mixed-model repeated measure analysis

^b^ P value for changes within groups from baseline to 3-months

^c^ P value for changes within groups from baseline to post-intervention
