# Supplementary material for: Effects of supervised aerobic exercise on cardiorespiratory fitness and patient-reported health outcomes in colorectal cancer patients undergoing adjuvant chemotherapy—a pilot study
Source: Support Care Cancer. 2021 Oct 8;30(3):1945–55. doi: 10.1007/s00520-021-06608-9 (PMC8795052; doi:10.1007/s00520-021-06608-9)
Supplement: Supplementary file 3 — Supplementary file3 (DOCX 34.0 KB) [file 520_2021_6608_MOESM3_ESM.docx]

**Effects of supervised aerobic exercise on cardiorespiratory fitness and patient-reported health outcomes in colorectal cancer patients undergoing adjuvant chemotherapy – a pilot study**

Supportive Care in Cancer

Eva M Zopf, Holger Schulz, Jonas Poeschko, Kerstin Aschenbroich, Thomas Wilhelm, Ernst Eypasch, Elmar Kleimann, Kai Severin, Jutta Benz, Enwu Liu, Wilhelm Bloch, Freerk T Baumann

Corresponding author: PD Dr Freerk T Baumann, Department of Internal Medicine, Center of Integrated Oncology Cologne Bonn, University Hospital of Cologne, Cologne, Germany, [freerk.baumann@uk-koeln.de](mailto:freerk.baumann@uk-koeln.de)

**Online resource 3** Cohen’s d effect sizes for changes between groups at 3-months and post-intervention

|  |  | Cohen’s d effect size for change between groups at 3-months | Cohen’s d effect size for change between groups post-intervention |
| --- | --- | --- | --- |
| VO2peak relative |  | - | 0.53 |
| Wattmax |  | - | 0.51 |
| HRmax |  | - | 0.17 |
| BMI |  | - | 0.06 |
| Weight |  | - | 0.08 |
| MFI |  |  |  |
| General fatigue |  | 0.23 | 0.17 |
| Physical fatigue |  | 0.28 | 0.11 |
| Reduced activity |  | 0.02 | 0.13 |
| Reduced motivation |  | 0.29 | 0.25 |
| Mental fatigue |  | 0.36 | 0.05 |
| EORTC-QLQ C30 |  |  |  |
| Global health status/QoL | | 0.03 | 0.09 |
| Physical function | | 0.19 | 0.06 |
| Role function | | 0.36 | 0.19 |
| Emotional function | | 0.22 | 0.03 |
| Cognitive function | | 0.01 | 0.07 |
| Social function | | 0.28 | 0.12 |
| Fatigue | | 0.20 | 0.01 |
| Nausea/vomiting | | 0.03 | 0.14 |
| Pain | | 0.01 | 0.01 |
| Dyspnea | | 0.24 | 0.02 |
| Insomnia | | 0.09 | 0.23 |
| Appetite loss | | 0.19 | 0.07 |
| Constipation | | 0.01 | 0.04 |
| Diarrhea | | 0.04 | 0.06 |
| Financial difficulties | | 0.26 | 0.07 |
| EORTC-QLQ CR29 | |  |  |
| Urinary frequency | | 0.37 | 0.11 |
| Blood and mucus in stool | | 0.52 | 0.42 |
| Body image | | 0.06 | 0.19 |
| Urinary incontinence | | 0.03 | 0.02 |
| Dysuria | | 0.02 | 0.05 |
| Abdominal pain | | 0.14 | 0.02 |
| Buttock pain | | 0.05 | 0.25 |
| Bloated feeling | | 0.09 | 0.06 |
| Dry mouth | | 0.08 | 0.01 |
| Hair loss | | 0.07 | 0.09 |
| Trouble with taste | | 0.09 | 0.27 |
| Anxiety | | 0.16 | 0.06 |
| Weight | | 0.11 | 0.01 |
| Flatulence | | 0.05 | 0.28 |
| Faecal incontinence | | 0.15 | 0.05 |
| Sore skin around anus or stoma | | 0.04 | 0.11 |
| Stool frequency | | 0.10 | 0.12 |
| Embarrassment by defaecation pattern or stoma | | 0.04 | 0.17 |
| Sexual interest (male) | | 0.13 | 0.29 |
| Impotence (male) | | 0.10 | 0.12 |
| Sexual interest (female) | | 0.07 | 0.15 |
| Dyspareunia (female) | | 0.04 | 0.08 |
| Freiburger Questionnaire of Physical Activity | | | |
| Leisure time & sport activity (h/week) | | 0.20 | 0.15 |

Abbreviations: BMI body mass index, EORTC-QLQ C30 European Organization for the Research and Treatment of Cancer Quality of Life Questionnaire, EORTC-QLQ CR29 colon cancer specific module, HRmax maximal heart rate, MFI Multidimensional Fatigue Inventory, QoL Quality of life, VO2peak peak oxygen uptake, Wattmax maximal work rate
